# Fabrication of Polypyrrole/Graphene Oxide Composite Nanosheets and Their Applications for Cr(VI) Removal in Aqueous Solution

**DOI:** 10.1371/journal.pone.0043328

**Published:** 2012-08-22

**Authors:** Shangkun Li, Xiaofeng Lu, Yanpeng Xue, Junyu Lei, Tian Zheng, Ce Wang

**Affiliations:** Alan G. MacDiarmid Institute, College of Chemistry, Jilin University, Changchun, People’s Republic of China; Queen’s University at Kingston, Canada

## Abstract

In this paper, we report on the simple, reliable synthesis of polypyrrole (PPy)/graphene oxide (GO) composite nanosheets by using sacrificial-template polymerization method. Herein, MnO_2_ nanoslices were chosen as a sacrificial-template to deposit PPy, which served as the oxidant as well. During the polymerization of pyrrole on surface of GO nanosheets, MnO_2_ component was consumed incessantly. As a result, the PPy growing on the surface of GO nanosheets has the morphology just like the MnO_2_ nanoslices. This method can provide the fabrication of PPy nanostructures more easily than conventional route due to its independence of removing template, which usually is a complex and tedious experimental process. The as-prepared PPy/GO composite nanosheets exhibited an enhanced properties for Cr(VI) ions removal in aqueous solution based on the synergy effect. The adsorption capacity of the PPy/GO composite nanosheets is about two times as large as that of conventional PPy nanoparticles. We believe that our findings can open a new and effective avenue to improve the adsorption performance in removing heavy metal ions from waste water.

## Introduction

The increased concern about global environment pollution problem has resulted in continuous expansion in finding available approaches to deal with heavy metal ions. Among those various kinds of heavy metal ions, Cr(VI) ion is considered to be one of the most toxic ions, which is found to be carcinogenic. The Cr(VI) ion has been extensively used in some polluted industries, like steel manufacturing, metal plating, military purposes, and leather tanning, as well as in the pigment and refractory industries [1], [2]. With the result of discharging a mass of Cr(VI) ions from industrial waste and sewage, people’s health and surrounding environment are in serious threat. Till now, several technologies have been developed to reduce/remove Cr(VI) ions from aqueous solutions such as adsorption, precipitation, ion exchange, membrane process and chemical coagulation [3]–[8]. Among these methods, adsorption is one of the most simple, effective and economically favorable way. However, the conventional adsorbents often showed a limited adsorption capacity because their surface area is not enough large. Therefore, there is still a requirement for new adsorbents exhibiting a high Cr(VI) removal capacity.

Intrinsically conducting polymers have been widely investigated in the past four decades due to their potential applications in nanoelectronic devices, energy conversion and storage devices, sensors, catalysis, electrochromic devices, actuators and biomedicine [9]–[13]. Among the conducting polymers, polypyrrole (PPy) has been most extensively studied for its facile synthesis, intriguing electronic and redox properties [14]–[17]. In addition to the investigated applications mentioned above just like other conducting polymers, PPy has also exhibited a good prospect in adsorption application for its positively charged nitrogen atoms in the polymer chains. Since Rajehwar et al. demonstrated for the first time that the PPy films could be used for reducing and removing Cr(VI) ions from waste water, the application of PPy materials dealing with the heavy metal ions was intensively investigated [18]. As we all know, one of the most important influencing factors to adsorption efficiency is the surface area or porosity of the adsorption media. Therefore, it is very necessary to prepare nanostructured PPy with a large surface area to improve its adsorption efficiency. On the other hand, active carbon is considered as one of the most widely applied adsorbents. As one of the important carbon family members, graphene oxide (GO) is believed to be one of the most interesting materials of this century [19]. GO nanosheet has a unique two-dimensional nature and contain a range of reactive oxygen functional groups on its surface, which renders them a good candidate for supporting other functional nanomaterials [20]–[22]. Recently, some work has been done on the preparation of PPy/GO and PPy/graphene nanocomposites for their applications in supercapacitor, transparent electrodes and environmental field [23]–[25]. In this case, PPy films or nanoparticles are deposited on the surface of GO or graphene nanosheets. However, there are few reports on constructing hierarchical PPy/GO nanosheets by combining 2D nanosheets of GO and 3D nanoflowers of PPy.

In this study, we have reported for the preparation of PPy/GO composite nanosheets by using a simple and reliable sacrificial-template polymerization method. First, MnO_2_ nanoslices were deposited on the GO nanosheets, which acted as both the template for shaping PPy nanostructure and oxidant source simultaneously. During the polymerization of pyrrole on surface of GO nanosheets, MnO_2_ component was consumed incessantly. As a result, the PPy growing on the surface of GO nanosheets has the morphology of nanoslices just like the MnO_2_ nanoslices. The as-prepared PPy/GO composite nanosheets exhibited an enhanced properties for Cr(VI) ions removal in aqueous solution based on the synergy effect. The adsorption capacity of the PPy/GO composite nanosheets is about two times as large as that of conventional PPy nanoparticles. We believe that our findings can open a new and effective avenue to improve the adsorption performance in removing heavy metal ions from waste water solution.

## Results and Discussion

The procedure for the fabrication of PPy/GO composite nanosheets involved three steps. First, GO was prepared by oxidizing graphite with acid and followed by ultrasonication. Then the exfoliated GO nanosheets were coated by MnO_2_ nanoslices layer by a following reaction: 4MnO_4_^−^+4H^+^ → 4MnO_2_+3O_2_+2H_2_O.

**Figure 1:**
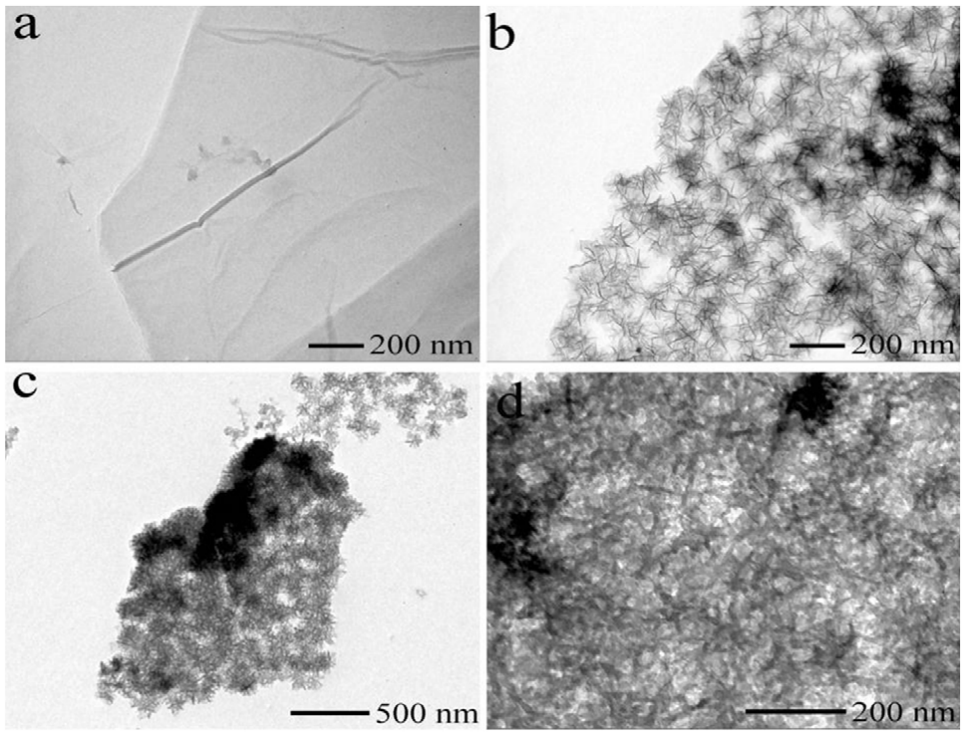
Instrumental analysis of morphology. (a) TEM image of GO, (b) TEM image of MnO_2_/GO nanocomposites, TEM images of PPy/GO composites nanosheets with (c) low and (d) high magnification.

At last, PPy nanoflowers were decorated on the surface of GO nanosheets by using MnO_2_ as a sacrificial template and the oxidant. The morphologies of the GO, as-prepared MnO_2_/GO nanocomposites and PPy/GO composite nanosheets were characterized by TEM images. As shown in Figure 1a, the as-synthesized GO is sheet in shape with a smooth surface. After growing MnO_2_ on the surface of GO, the GO nanosheet is covered with nanoslices of MnO_2_. The MnO_2_ nanoslices with a scale in the range of 30–80 nm were aggregated to form a flower like morphology. It is well known that the oxidant potential of MnO_2_ is much higher than that of the pyrrole polymerization. Therefore, PPy would be polymerized on the surface of MnO_2_ when we mixed the as-prepared MnO_2_/GO nanocomposites and pyrrole monomer. During the polymerization, the oxidant of MnO_2_ was consumed, resulting in PPy/GO composite nanosheets. Figure 1c displays the TEM image of the obtained PPy/GO composite nanosheets. We can easily find that the PPy/GO composite nanosheets generally reserve the morphologies of MnO_2_/GO nanocomposites. The nanoflower-like PPy was synthesized and well covered on the surface of GO nanosheets. Taking TEM image with a relatively high magnification, more details about the morphology of the nanocomposites are observed, in which the nanoflower-like PPy nanomaterials were also composed of many slices. This result proved that the formation of PPy/GO composite nanosheets was using MnO_2_/GO nanocomposites as templates.

**Figure 2:**
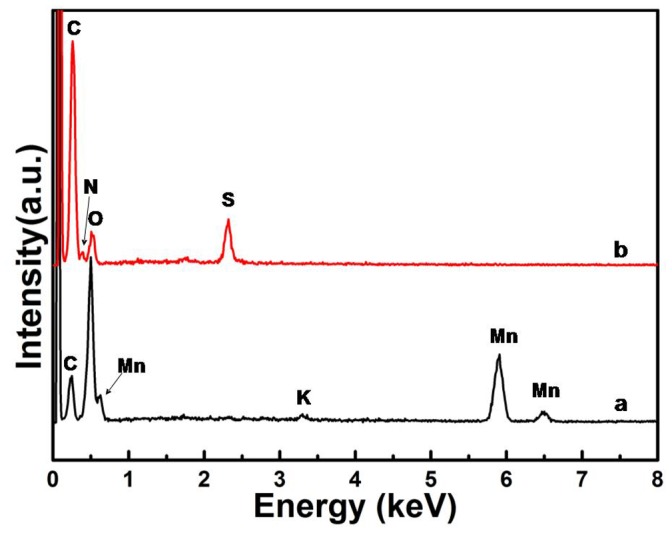
EDX spectra of (a) MnO_2_/GO nanocomposites, (b) PPy/GO composite nanosheets.

The formation of the resulting MnO_2_/GO nanocomposites and PPy/GO composite nanosheets has been proved and characterized by EDX, FTIR spectra, and XRD measurements. Figure 2a shows the EDX spectrum of the prepared MnO_2_/GO nanocomposites, which reveal the presence of C, Mn, and O elements in the samples. It also shows a small amount of K in the sample, which is owing to the intercalation of K^+^ between the layered structure of MnO_2_. Figure 2b shows the EDX results of the as-prepared PPy/GO. It was clearly observed that Mn element has been completely disappeared, implying that the MnO_2_ was removed after the polymerization of pyrrole monomer. The characteristic elements of GO and PPy, such as C, O, N and S were observed, indicating the formation of the PPy/GO composite nanosheets. The element of S should be originated from the doped acid of H_2_SO_4_ in the PPy.

**Figure 3:**
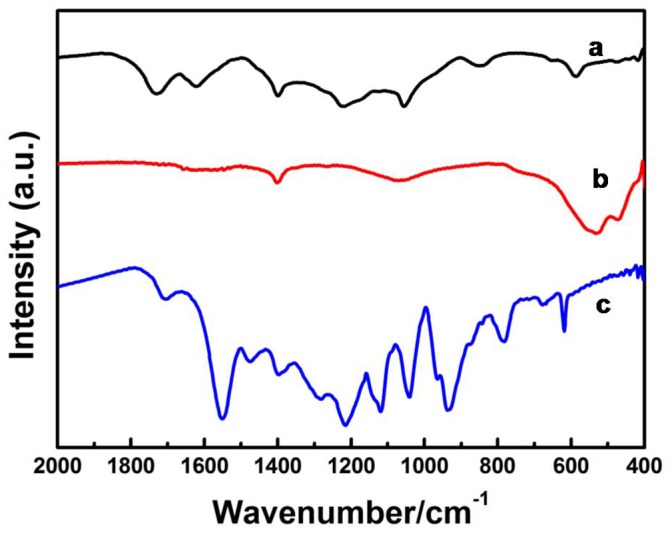
FTIR spectrum of (a) GO, (b) MnO_2_/GO nanocomposites and (c) PPy/GO composite nanosheets.

The FTIR spectrum of the GO, MnO_2_/GO nanocomposites and PPy/GO composite nanosheets are present in Figure 3. In the FTIR spectrum of GO, the characteristic peak at 1730 cm^−1^ can be ascribed to C = O stretching vibration. The peaks at 1056 and 1220 cm^−1^ are ascribed to alkoxy C-O and epoxy C-O stretching vibrations, respectively. The peak at 1400 cm^−1^ is attributed to the O-H deformation vibration. The band at 1621 cm^−1^ is probably due to the adsorbed water. These results are similar with the previous reports [26], [27]. Figure 3b shows the FTIR spectrum of MnO_2_/GO nanocomposites, the strong peaks at 535 cm^−1^ are assigned to the Mn-O vibration of MnO_2_ products. As the peak from Mn-O at 535 cm^−1^ is too strong, the peaks of GO are concealed, most of them can hardly be observed from the spectrum. From the FTIR spectrum of PPy/GO (Figure 3c), we can find the peak at 535 cm^−1^ is disappeared which imply the MnO_2_ were all removed after the MnO_2_ reacting with pyrrole monomer. Other characteristic bands in the spectra can be attributed to both PPy and GO. The peaks at 1551 and 1474 cm^−1^ are assigned to the antisymmetric and symmetric pyrrole-ring fundamental vibration of PPy, respectively. The peaks at 1281 and 1215 cm^−1^ are related to C-N stretching vibration and C-C stretching vibration, respectively. The peaks at 934 and 783 cm^−1^ are attributed to the C-H out-of-plane vibration of PPy. These characteristic bands indicate the formation of PPy [28], [29]. In addition, the characteristic peak assigned to C = O and C-O stretching vibration from GO has been shifted to 1703 and 1041 cm^−1^, respectively, which is probably attributed to the interactions between GO and PPy rings [26].

**Figure 4:**
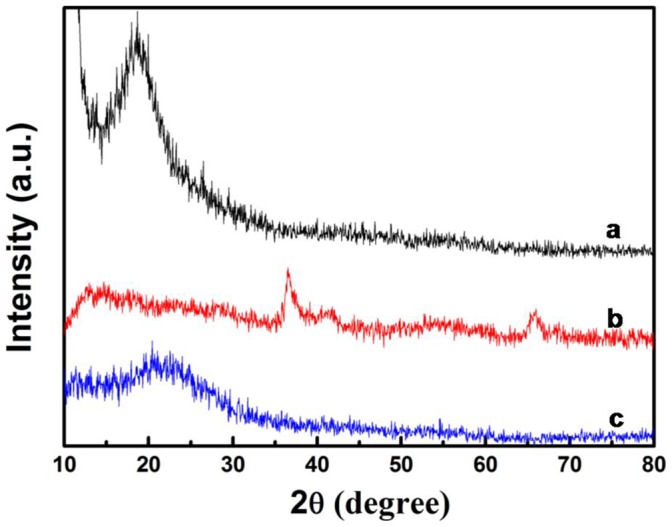
The XRD analysis of the samples. (a) XRD pattern of GO, (b) XRD pattern of MnO_2_/GO nanocomosites and (c) XRD pattern of PPy/GO composite nanosheets.

Figure 4 shows the XRD patterns of the GO, MnO_2_/GO nanocomposites and PPy/GO composite nanosheets. The XRD patterns of MnO_2_/GO is shown in Figure 3b, compared with GO (Figure 4a), the diffraction peaks at 2θ values of 36.5, 41.7 and 66.0° are observed. These peaks could be ascribed to the (211), (301) and (002) crystal planes of α-MnO_2_ [30], [31]. However, these peaks are broadened, which indicates the poor crystallinity of MnO_2_ in MnO_2_/GO nanocomposites. After the formation of PPy/GO composite nanosheets, the peaks of MnO_2_ disappeared, indicating that the MnO_2_ were all removed (Figure 4c). XRD patterns also reveals that the PPy/GO composite nanosheets obtained in this study are essentially amorphous, since a broad peak appears at 2θ = 22.4°, which can be attributed to amorphous PPy and GO.

**Figure 5:**
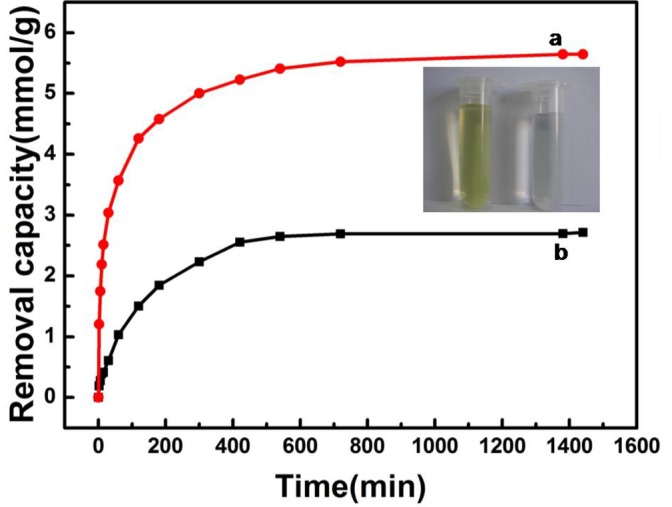
Comparison of the adsorption properties. The removal capacity of (a) the PPy/GO and (b) conventional PPy nanoparticles for Cr(VI) ions (0.85 mM) in the aqueous solution at different time. A photograph representing the outstanding removal ability of PPy/GO composite nanosheets for Cr(VI) ions (0.85 mM).

The PPy/GO composite nanosheets with flower-like PPy nanostructures afford its potential applications for waste water treatment. In this study, the feasibility of the PPy/GO as a Cr(VI) ions remover was explored. The concentration of Cr(VI) ions in aqueous solution was measured by UV-vis spectroscopy. Base on the calibration curve, the removal amount of Cr(VI) ions can be calculated. Figure 5 shows the removal capacity of Cr(VI) ions (0.85 mM) in the aqueous solution at different time. The removal capacity of the PPy/GO composite nanosheets and conventional PPy nanoparticles was compared. As shown in Figure 5, the equilibrium removal capacity of Cr(VI) ions for PPy nanoparticles and PPy/GO was 2.715 and 5.644 mmol/g, respectively. The removal capacity of PPy/GO composite nanosheets was two times as large as that of conventional PPy nanoparticles. To evaluate the effect of surface area on the adsorption properties, the nitrogen adsorption and desorption isotherm techniques was used to test Brunauer–Emmett–Teller (BET) surface areas of PPy/GO composite nanosheets and conventional PPy nanoparticles, which was 84.8 and 41.3 m^2^/g, respectively. This result indicates the larger removal capacity of Cr(VI) ions by PPy/GO composite nanosheets is strongly related to their large surface area. From the above results, it could be concluded that the PPy/GO composite nanosheets had excellent potential applications in Cr(VI) ion removal. The photograph in Figure 5 shows the changes of Cr(VI) ions solution before and after adsorption by PPy/GO composite nanosheets. The color of the initial Cr(VI) solution was light yellow (left-hand side), and after addition of the PPy/GO composite nanosheets it turned almost transparent (right-hand side).

**Figure 6:**
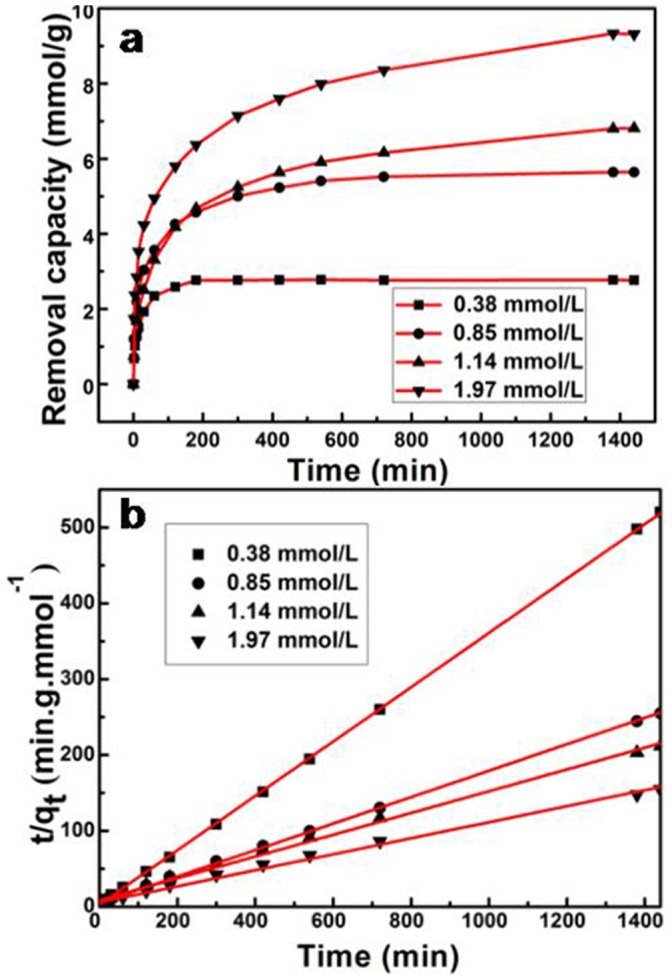
The effect of the Cr(VI) initial concentration on the Cr(VI) removal. (a) The removal capacity of PPy/GO for different concentration of Cr(VI) ions with time. (b) The pseudo-second-order model for adsorption of Cr(VI) by the PPy/GO composite nanosheets.

We also studied the effect of the Cr(VI) initial concentration on the Cr(VI) removal by the PPy/GO composite nanosheets (Figure 6a). It was found that the equilibrium will be reached in 180 min when the initial concentration of Cr(VI) was 0.38 mmol/L. With increasing the Cr(VI) initial concentration to 1.97 mmol/L, it requires about one day for equilibrium to be reached. On the other hand, the adsorption efficiency (percentage removal) is also strongly dependent to the initial concentration of Cr(VI). The adsorption efficiency decreased from 100% to 70.8% when the initial concentration of Cr(VI) varied from 0.38 mmol/L to 1.97 mmol/L. This may be owing to the rigorous competition for the adsorption sites with increasing the concentration of Cr(VI), which is consistent with the previous report [32]. The kinetics of Cr(VI) removal on the PPy/GO composite nanosheets was also investigated at various concentrations of Cr(VI). Figure 6b shows the pseudo-second-order model for adsorption of Cr(VI) by the PPy/GO composite nanosheets. The linearized form of the pseudo-second-order equation is as follows:
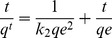
Where q_e_ is the equilibrium Cr(VI) removal capacity and q_t_ is Cr(VI) removal capacity at time t. k_2_ is the pseudo-second-order rate constant. From the plots t/q_t_ vs. t of the PPy/GO adsorbent at initial concentrations of Cr(VI) varied from 0.38 to 1.97 mmol/L, the pseudo-second-order rate constant (k_2_) decreases from 0.0452 to 0.0019 g/mmol/min. The calculated q_e_ is also close to the experimental one and the correlation coefficient (R^2^) is above 0.994. This result indicates that the adsorption kinetics of the Cr(VI) removal by the PPy/GO composite nanosheets follows the pseudo-second-order model, which is based on the assumption of the chemisorption process [33]–[36].

**Figure 7:**
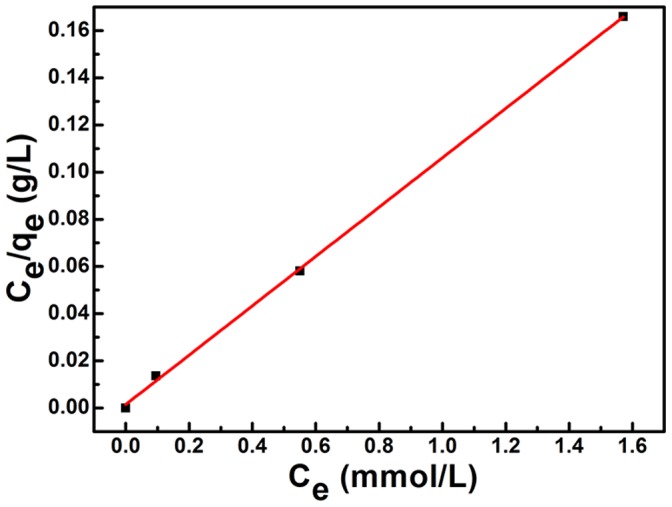
Fit of equilibrium data to Langmuir isotherm model.

Furthermore, the Langmuir model has been used to evaluate the adsorption performance of Cr(VI) on PPy/GO composite nanosheets, which could be written as follows [37]:
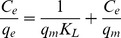
where q_m_ stands for the maximal adsorbent capacity of Cr(VI) ions on the adsorbent (mmol/g) and K_L_ is the adsorption equilibrium constant (L/mmol). The values of q_m_ and K_L_ can be calculated from the values of the slope and the y-intercept of the plot. Figure 7 displays the relationship between C_e_/q_e_ and C_e_, indicating that the adsorption data of Cr(VI) ions on PPy/GO composite nanosheets were fitted particularly well with the Langmuir model (the correlation coefficient R^2^ = 0.999). The maximal adsorption capacity of Cr(VI) ions by PPy/GO composite nanosheets was about 9.56 mmol/g.

A comparison of maximum adsorption capacity between PPy/GO composite nanosheets and other PPy based nanomaterials reported in literature has been presented in Table 1. It can be seen that the adsorption capacity of the PPy/GO composite nanosheets is much higher than those materials [38]–[41]. The high adsorption capacity of Cr(VI) ions on PPy/GO composite nanosheets make them excellent potential applications in industrial waste water treatment.

**Table 1:** Comparison of adsorption capacity of the PPy/GO nanocomposites with other adsorbents for Cr(VI) removal.

Adsorbents	q_m_ (mg/g)	Equilibriumtime (h)	Optimum pH	Ref.
Polypyrrole/wood sawdust	3.4	0.16	5.0	[38]
Polypyrrole/Fe_3_O_4_ magnetic nanocomposite	243.9	3	2.0	[39]
Orange-like Fe_3_O_4_/PPy composite microspheres	209.2	12	2.0	[40]
Polypyrrole-polyaniline nanofibers	227	3	2.0	[41]
Polypyrrole/graphene oxide composite nanosheets	497.1	24	3.0	[Present study]

**Figure 8:**
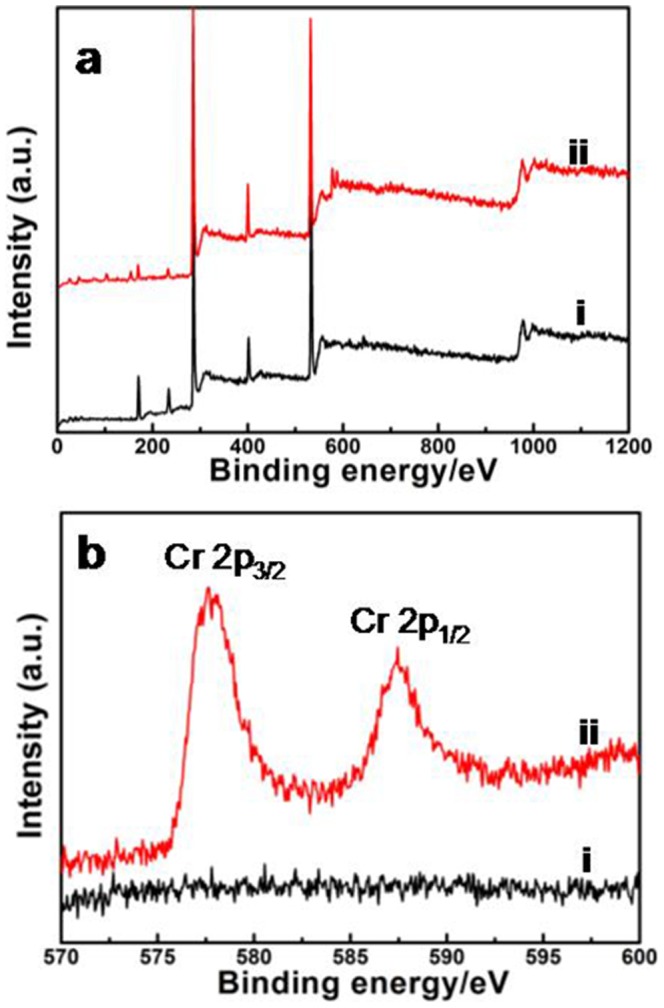
The XPS spectra of samples. (a) The XPS spectra of PPy/GO composite nanosheets i) before and ii) after adsorbing Cr(VI) ions. (b) The XPS spectra of Cr in samples i) before and ii) after adsorbing Cr(VI) ions.

To investigate the mechanism of Cr (VI) adsorption by PPy/GO composite nanosheets, the XPS spectra of the PPy/GO composite nanosheets before and after adsorption of Cr(VI) ions was given in Figure 8. Compared to the PPy/GO composite nanosheets before adsorption of Cr(VI) ions, two energy bands at about 577.5 eV and 587.2 eV corresponding to the binding energies of Cr(2p_3/2_) and Cr(2p_1/2_) are observed after adsorption. The presence of Cr(2p_3/2_) proves the existence of the oxidation state of Cr(III). This result suggests that both Cr(III) and Cr(VI) were existing on the surface of the PPy/GO composite nanosheets after their adsorption of Cr(VI) ions. It is easily understood the existence of Cr(VI) species on the surface of PPy/GO composite nanosheets is owing to the adsorption of Cr(VI) ions through the anion exchange property of PPy by replacing the doped SO_4_^2−^ ions. However, the presence of Cr(III) on the surface of adsorbents indicates that some fraction of adsorbed Cr(VI) was reduced to Cr(III) during the adsorption process. The reduction phenomena might be due to the presence of positive nitrogen group in PPy in the nanocomposites.

## Materials and Methods

### Chemicals

Graphite powder was purchased from Sinopharm Chemical Reagent Co., Ltd. Pyrrole monomer was purchased from Aladdin and stored at −4°C prior to use; and all other chemicals (H_2_SO_4_, KMnO_4_, K_2_Cr_2_O_7_) were obtained from Beijing Chemical Corporation. The chemicals were analytical grade and used without further purification.

### Preparation of MnO_2_/GO Nanocomposites

GO was synthesized based on the modified Hummers and Offeman’s method [42], [43]. In a typical preparation of MnO_2_/GO nanocomposites, 30 mg of GO was dispersed in 26 ml H_2_O by ultrasonication. Then 3 ml of concentrated H_2_SO_4_ were added into the solution by ultrasonication. After the solution was heated at 90°C for 5 min, 150 mg of KMnO_4_ was added into the solution under magnetic stirring at 90°C. After vigorous stirring for 20 min, the MnO_2_/GO nanocomposites were obtained by centrifuging and washed with water for three times and dried at 40°C in vacuum for one night.

### Preparation of PPy/GO Composite Nanosheets

75 mg of MnO_2_/GO composites was dispersed in 50 ml H_2_O by ultrasonication, then 125 µL of pyrrole monomer was added into the above solution (The thermogravimetric analysis (TGA) gives that the weight content of GO in MnO_2_/GO nanocomposites was about 16%. Combined with the added content of the MnO_2_/GO and pyrrole monomer, we can calculate that the weight ratio between GO and pyrrole monomer was about 1∶10). 2.5 ml concentrated H_2_SO_4_ were added into the above solution under magnetic stirring. After 2 h, the PPy/GO composite nanosheets were obtained by centrifuging and washed with water for three times.

The conventional PPy nanoparticles were prepared with the previous report [44].

### Application of As-prepared PPy/GO Composite Nanosheets for Cr (VI) Ions Removal

The Cr(VI) solution was prepared by potassium dichromate (K_2_Cr_2_O_7_) at pH = 3.0. In order to determine the concentration of Cr (VI) ions a calibration curve of Cr(VI) ion was made by UV-Vis spectrophotometer. The calibration curve was obtained from standard solutions of Cr(VI) (0.2–1.2 mmol/L) solution.

For the effect of Cr(VI) concentration of solution on the removal capacity experiment, the as-prepared PPy/GO composite nanosheets powder (3 mg) was dispersed into 20 mL of Cr(VI) solution with different concentrations at pH = 3. After reaction for 24 h, the reaction solution after magnetic stirring for a certain time, the solution was extracted for ultraviolet analysis to determine its concentration. Based on calibration curve, the removal amount of Cr(VI) ions could be calculated. The removal capacity of Cr(VI) ions could be calculated by the following formula.
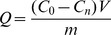
Where V(L) is the initial volume of Cr(VI) solution, m (g) is the mass of the as-prepared PPy/GO, C_0_ (mmol/L) is the initial concentration of Cr(VI) ions, C_n_(mmol/L) is the n times extracted solution’s concentration, Q (mmol/g) for the removal or reduction capacity, respectively.

### Characterization

The morphology and composition of the products were characterized by TEM (JEM-1200EX) and EDX analyses (SSX-550, Shimadzu). FT-IR spectra were recorded on a BRUKER VECTOR22 Spectrometer using KBr pellets. XRD patterns were obtained with a Siemens D5005 diffractometer with CuKα radiation and SmartLab goniometer. Analysis of the X-ray photoelectron spectra (XPS) was performed on Thermo ESCALAB 250 spectrometer with a Mg-K (1253.6 eV) achromatic X-ray source. The Cr(VI) ions solution were recorded using SHIMADZU UV−2501 UV-vis spectrophotometer.
